# Environmental Sustainability Assessment of Dairy Farms Rearing the Italian Simmental Dual-Purpose Breed

**DOI:** 10.3390/ani10020296

**Published:** 2020-02-13

**Authors:** Mario Baldini, Francesco Da Borso, Andrea Rossi, Mario Taverna, Stefano Bovolenta, Edi Piasentier, Mirco Corazzin

**Affiliations:** 1 Department of Agricultural, Food, Environmental and Animal Sciences, University of Udine, 33100 Udine, Italy; mario.baldini@uniud.it (M.B.); francesco.daborso@uniud.it (F.D.B.); mario.taverna@uniud.it (M.T.); stefano.bovolenta@uniud.it (S.B.); edi.piasentier@uniud.it (E.P.); 2 La Rossa Pezzata of Friuli Venezia Giulia cooperative society, 33080 Fiume Veneto, Italy; andrearossi1992@gmail.com

**Keywords:** cattle, LCA, climate change, acidification, eutrophication

## Abstract

**Simple Summary:**

The milk and meat production systems are responsible for more than 5% of greenhouse gas emissions in the world; therefore, there is a strong need to propose strategies for reducing the carbon footprint. The aim of this paper was to assess the environmental footprint of dairy farms rearing a dual-purpose breed and to evaluate the fattening of calves directly in farms and the cultivation of alternative crops, such as hemp, as possible strategies for reducing the environmental footprint of dairy farms. In order to produce 1 kg of milk with 4.0% of fat and 3.3% of protein, the emissions were 1.1–1.4 kg CO_2_eq, 14.8–19.0 g SO_2_eq and 5.0–6.4 g PO_4_^3−^eq. These emissions could be reduced by 8–11% by fattening the calves directly in the farms, and by 1–5% by cultivating hemp and using its by-product, hempseed cake, in the diets of animals. Moreover, the results of this study showed that the environmental footprint can be reduced, improving the productive efficiency of the farms.

**Abstract:**

This study aimed to assess the environmental footprint of dairy farms rearing a dual-purpose breed, and to evaluate, through alternative scenario analyses, the fattening of calves and the cultivation of hemp as strategies for reducing the environmental impact of these farms. Eleven farms were evaluated for global warming (GWP), acidification (AC) and eutrophication (EUP) potential. The Life Cycle Assessment method with three scenarios, REAL, based on real data, BEEF, where calves were fattened in farm, and HEMP, where hemp was cultivated in farms, were considered. If referred to 1 m^2^ of utilizable agricultural land, the GWP, AC and EUP were 1.6 kgCO_2_eq, 21.7 gSO_2_eq and 7.1 gPO_4_^3−^eq, respectively. If referring to 1 kg of fat and protein corrected milk, the emissions were 1.1–1.4 kgCO_2_eq, 14.8–19.0 gSO_2_eq, and 5.0–6.4 gPO_4_^3−^eq, depending on the allocation method adopted. The emissions were associated positively with culling rate and negatively with production intensity. In BEEF and HEMP scenarios, the emissions were reduced by 8–11% and by 1–5%, respectively. Fattening the calves, evaluating the cultivation of alternative plants and improving the productive and reproductive efficiency of animals could be effective strategies for reducing the environmental footprint of the farm.

## 1. Introduction

The livestock sector contributes to about 15% of all emissions of greenhouse gases (GHG), and more than half of these emissions derive from milk and meat production systems [1]. Despite this important environmental impact, it must be considered that the survival of over one billion people, concentrated mainly in developing countries, depends on livestock rearing [2]. Furthermore, about 20% of the land surface free from ice is made up of grasslands [3] and, in this environment, ruminants can play an important role, being able to convert a food unsuitable for human consumption, forage, into high nutritional value foods such as meat and milk. White and Hall [4], in a simulation study, revealed that eliminating animal husbandry in the USA would result in a reduction of GHG emissions close to 30%, but at the same time, the agricultural system would not be able to completely satisfy the nutritional needs of Americans. Therefore, it is important not only to quantify but also to understand what strategies can be used to reduce the environmental footprint of products from ruminants.

Italian Simmental (IS) is a dual-purpose cattle breed belonging to the Simmental population, that, with more than 40 million animals, is one of the most important cattle breeds in the world. In Italy, in 2018, about 5% of all the dairy cows under functional control were Italian Simmental [5]. This breed has a greater efficiency of producing meat and lean tissue than dairy breeds [6], and therefore, from an environmental point of view, the total emissions related to milk production can also be significantly apportioned to beef, potentially reducing the environmental footprint per kg of milk produced on a physical basis.

Hemp (*Cannabis sativa* L.) is a bioenergetic crop that can be used for the production of bioethanol, biogas and combustion biomass [7]; moreover, the reproductive and vegetative organs are rich in secondary metabolites, such as cannabinoids, terpenoids and flavonoids [8], which have various applications in pharmacy, cosmetics, and as bio-pesticides and antimicrobials [9]. Currently, more than 90,000 ha are cultivated for hemp production in the world; thanks to the applications previously described and to the development of cultivars with low concentrations of cannabinoids, the worldwide market of products derived from this plant is expected to double in the next few years [10]. Moreover, hemp is considered a high yield and a low-input crop in the use of non-renewable resources because of its ability to minimize pesticide interventions and its low demand for fertilizers [11,12]. The hempseed cake, which is a by-product of hempseed obtained after the mechanical extraction of the oil, appears particularly interesting for animal nutrition. Indeed, it has a high biological value of protein, with an amino acid profile comparable to that of egg white and soybean [13]. Therefore, hemp by-products could potentially be used in dairy cattle diets and could contribute to increasing the environmental sustainability of a farm.

As reviewed by Baldini et al. [14], several studies were recently performed for evaluating the environmental footprint of dairy farms with the Life Cycle Assessment (LCA) [15], which is widely considered as a leading method for assessing the environmental impact of a product or of a production process, taking into account the entire life cycle. Indeed, this methodology was successfully applied for assessing the environmental footprint of milk production not only from cattle, but also from other species such as buffalo [16], and under different production systems [17,18,19,20]. However, fewer studies considering alternative crops or the possibility of fattening the calves of dual-purpose breeds directly in the farms are available [21,22]. In particular, the environmental assessment of hemp for fiber or ethanol production and for building applications was performed [23,24,25]; conversely, there is no information about the environmental impact of the use of hempseed in cattle feeding. 

The aim of this study was to assess the environmental footprint of dairy farms that rear a dual-purpose breed with an LCA approach, and to evaluate, through alternative scenario analyses, the fattening of calves, the cultivation of hemp and the use of its byproduct, hempseed cake, in animal diets as possible strategies for reducing the environmental footprint.

## 2. Materials and Methods

### 2.1. Goal, Scope Definition, System Boundaries and Functional Units

The environmental footprints were assessed through the LCA method [15,26,27,28,29]. In the present study, the goal of the LCA was to assess the environmental footprint of milk production in dairy farms that rear a dual-purpose breed, Italian Simmental, and to assess how the enhancement of the co-product beef and the cultivation of hemp and the use of the hempseed cake in the diet of animals can influence the emissions apportioned to milk yield and to the utilizable agricultural land (UAL).

**Figure 1:**
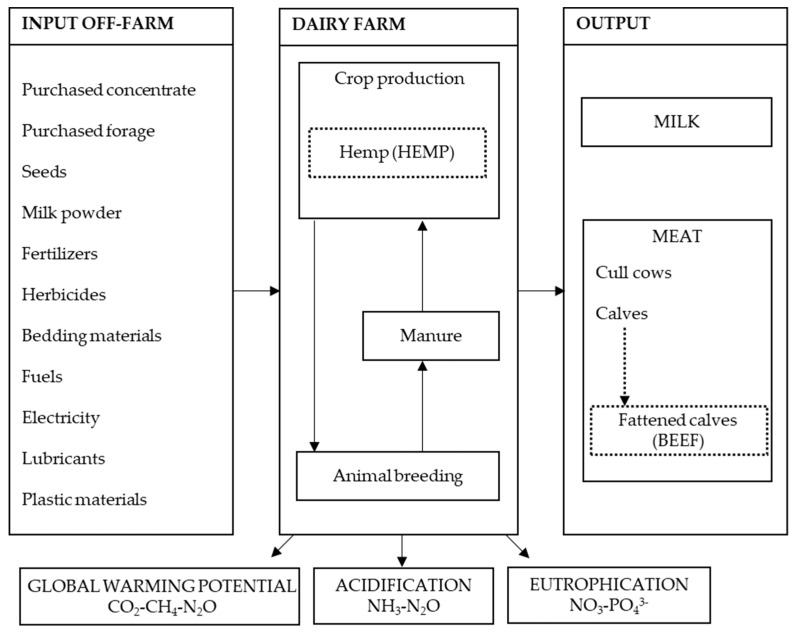
System boundaries diagram of Life Cycle Assessment applied to dairy farms. The dotted lines represent the alternative scenarios considered in the study.

The environmental footprints of the farms were assessed with a “cradle to farm gate” approach. Both the emissions related to the farm’s activities, on-farm, and those related to the inputs coming from outside the farm, off-farm, were considered. The farm buildings, machinery and the medicines for animals were not considered [30]. The study flow is presented in Figure 1.

The functional units considered were: 1 kg of fat and protein corrected milk (FPCM) [31] produced by cows and leaving the farm gate, and 1 m^2^ of UAL.

### 2.2. Data Collection, Inventory Analysis, Software and Impact Categories

Eleven dairy farms rearing the Italian Simmental breed located in Northern Italy were considered. The data were collected through a field investigation, farm balance sheets and financial documents such as invoices. Information about milk production and composition, and about herd composition, were obtained by the Italian Breeders Association. Moreover, data were also collected through a questionnaire administered to farmers regarding information about herd management, the housing system and manure management. The main characteristics of the dairy farms are reported in Table 1. The average number of animals reared per farm, expressed as the equivalent of one adult dairy cow (LU) [32], was 125. The dairy cows were mainly housed in cubicles; conversely, heifers and calves were loose housed, with straw or sawdust as bedding material. In all the farms, the manure was spread in their own fields that were within 10 km of distance. In three farms, the animals, with the exception of calves, had access to an external pasture for an average of 134 ± 24 d per year (mean ± se). As reported in Table 1, on average, around one third of the UAL was represented by meadows and pasture, 12% was cultivated with maize and sorghum for silage and 41% was used for other forage production (mainly alfalfa, 31%), while the remaining part, around 13% of the UAL, was cultivated almost entirely for cereals production.

The methane (CH_4_) and nitrous oxide (N_2_O) emissions were calculated according to Intergovernmental Panel on Climate Change (IPCC) [33], following the Tier 2 approach as reported in Salvador et al. [22] with updated conversion factors [34]. In order to assess N_2_O emissions, the compositions of the animals’ diets, and the protein content in particular, were assessed by chemical analysis—the Kjeldahl method [35]—and on the basis of data provided by commercial producers. The fuel and electricity used were obtained from the invoices. The estimation of emissions of ammonia (NH_3_), NO_x_ from fuel and electricity consumption, the N leaching at field level in the form of NO_3_^−^ and the phosphorus loss as PO_4_^3−^, as well as the manure phosphorus content, were assessed as reported in Salvador et al. [22].

The estimation of the off-farm emissions was performed with SimaPro 8.5 software [36] and by considering the Ecoinvent v. 3.4 [37] database. The CML-IA (Centre for Environmental Studies, University of Leiden, The Netherlands) baseline V3.05 method [38] updated in 2016 was used. 

The impact categories [34] considered were: Global warming potential (GWP), expressed as kg CO_2_eq factors in a 100-year time horizon; acidification potential (AC), expressed as g SO_2_eq; and eutrophication potential (EUP), expressed as g PO_4_^3−^eq. These categories are among the most frequently considered impact categories in the livestock sector [39].

### 2.3. Environmental Footprint Assessment, Alternative Scenario Analysis and Allocation Methods

As is usual for Italian dairy farms, the farms considered would sell the calves exceeding the culling rate a few weeks after birth. However, considering that they rear a dual-purpose breed, Italian Simmental, the fattening of calves directly on the farm could be a mitigation strategy, as well as the cultivation of a low-input and high-yield crop such as hemp. Therefore, three scenarios were considered in this study:

- BASE Scenario—in this scenario, the environmental footprint of the farms was assessed considering the real data.
- BEEF Scenario—in this scenario, it was hypothesized that the calves, instead of being sold at a few weeks of age, were fattened in farms until they reached the usual live weight for slaughtering for the IS breed, which is 620 kg and 480 kg for young bulls and heifers, respectively. The animals’ performance, diets and management were in accordance with Corazzin et al. [40] who took into account the IS breed. For the fattening of heifers, the same ingredients of the diets and the same management of young bulls were considered, while the animals’ performances were estimated according to IPCC [33]. Apart from the diet ingredients, other off-farm data for animals fattening, such as fuel and electricity consumptions, were taken into account, in accordance with Augusti et al. [41], who considered husbandry systems similar to those adopted in the farms of the present study.
- HEMP Scenario—in this scenario, it was considered that farms would cultivate enough hemp in an additional area to be able to include the hempseed cake in the animals’ diets at a level of 5% DM, which corresponds to the maximum level recommended by EFSA [42]. This resulted in an increase in the cultivated area of 76.5 ha, on average. The purchase of corn and soybean were adjusted in order to maintain the same energy and protein levels of the animals’ diets, as in the BASE Scenario. The data for hemp cultivation, field residues, biomass and hempseed cake production were taken according to Baldini et al. [43] and Amaducci et al. [44,45]. The apportionment of emissions from hempseed to hempseed cake was performed using the economical allocation; specifically, the allocation ratio was 80:20 hempseed oil/hempseed cake.

For each scenario, three allocation methods were considered:

- No allocation—all the emissions of the farm were allocated to FPCM and to UAL.
- Physical allocation—all the emissions were allocated not only to FPCM, but also to the farms’ co-products (calves and cull cows), as reported by IDF [30]. Consequently, the allocation ratios of milk/beef were 79:21, 64:36 and 79:21 in the BASE, BEEF and HEMP scenarios, respectively.
- Economic allocation—all the emissions were allocated to FPCM, calves and culls cows on the base of the real farm income, e.g., the FPCM allocation factor was calculated as the ratio between the total income from the milk sold and the total income of the farm. For the BEEF Scenario, the market prices for animals of the same breed, Italian Simmental, and the live weight at slaughter were considered [46]. The allocation ratios of milk/beef were 86:14, 78:22 and 86:14 in the BASE, BEEF and HEMP Scenarios, respectively.

### 2.4. Statistical Analysis

The assessment of the environmental footprints was carried out for each farm and the means are reported in Tables and Figures. The statistical analysis was performed using R software, version 3.4.0, [47] and the FactoMineR package [48]. In order to explore the relationship between the variables and the overall data variability, the farm characteristics and environmental footprints were subjected to Principal Component Analysis (PCA). In order to avoid redundancy, variables with a correlation higher than 0.9 were not considered in this analysis, as suggested by Tabachnick and Fidell [49]. 

## 3. Results and Discussion

### 3.1. Environmental Sustainability and Allocations in the Dairy Farms Rearing Dual Purpose Breed (BASE Scenario)

**Figure 2:**
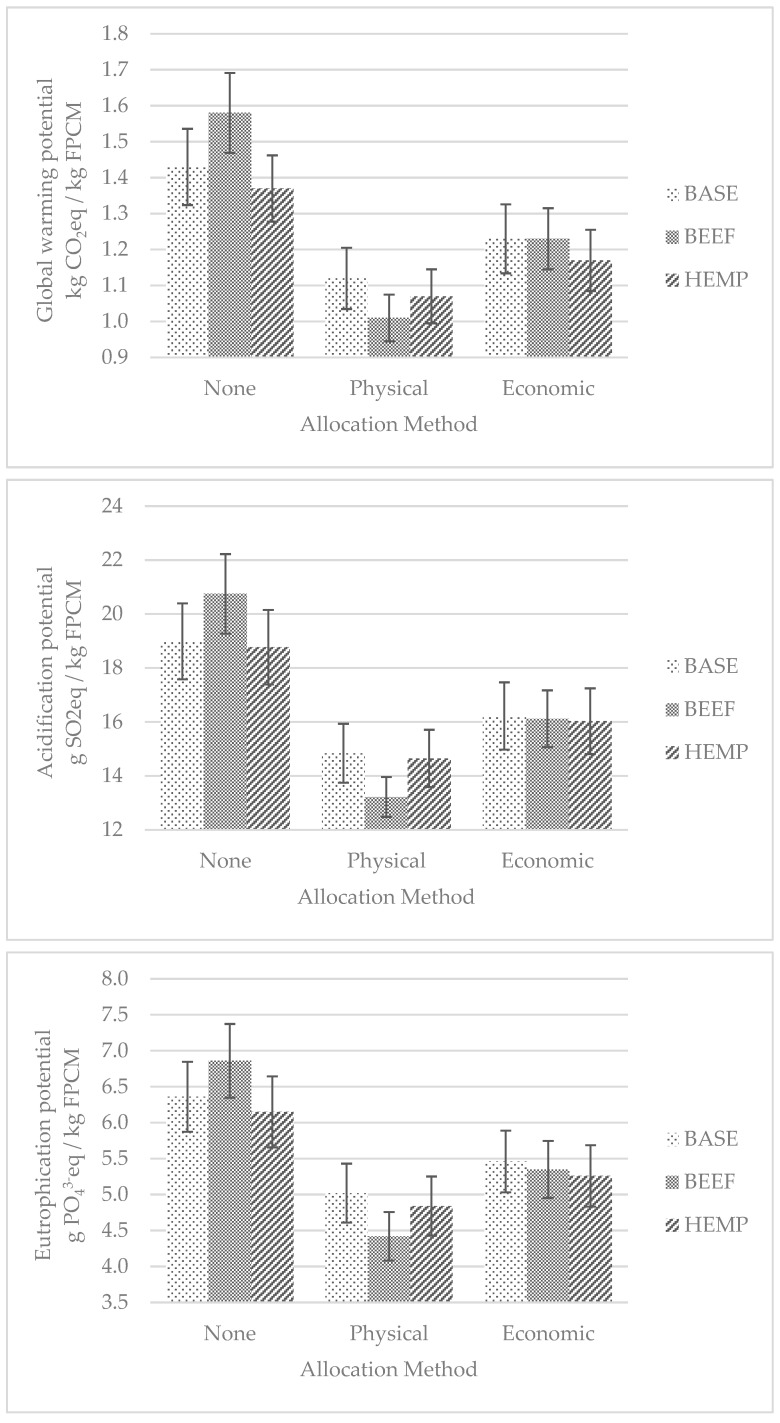
Environmental footprint of dairy farms rearing dual-purpose breed with different allocations, considering baseline scenario (BASE), milk-beef production system scenario (BEEF), the use of hempseed cake in the animals’ diets scenario (HEMP). Data are shown as mean ± se.

Figure 2 shows the values relating to the environmental footprint per kg FPCM of the farms in the three scenarios previously described. Considering the GWP without allocation and in the BASE scenario, the emission of CO_2_eq per kg of FPCM was 1.43 ± 0.106 kg, while the emission of CO_2_eq per m^2^ of UAL was 1.64 ± 0.130 kg (data not shown). These values are very similar to those reported by Thomassen et al. [50] who, considering 10 Dutch farms that had an average consistency of 81 lactating cows with a production of about 8 t per animal, obtained an average value of 1.40 kg of CO_2_eq/kg FPCM. These emissions values were recently confirmed by the study of Lovarelli et al. [18], where the average value of 1.46 kg CO_2_eq/kg FPCM was calculated for farms whose milk was used for the production of Parmigiano Reggiano cheese. Interestingly, the farms analyzed in Lovarelli et al. [18] had a milk yield per cow of 25.0 kg FPCM/d and a feed efficiency of 1.15 kg FPCM/kg DMI, similar to that of the farms considered in the present study. In the study of Guerci et al. [51], five Danish, two German and five Italian farms, with very variable conditions in terms of herd size, from 36 to 350 cows bred, in terms of production potential, from 6.2 to 10.9 t of milk per animal, and in terms of agricultural area, from 21 to 225 ha, were considered. In this study, the emissions in terms of CO_2_eq per kg of energy-adjusted milk (ECM) ranged widely from 0.55 to 1.91. However, if only the Italian farms were taken into account, the emissions ranged between 1.11 and 1.91 kg CO_2_eq/kg FPCM, with the highest value being observed in the farm and with the lowest production level per cow and the lowest feed efficiency. Salvador et al. [22], Guerci et al. [52] and Kiefer et al. [21] showed that the highest emission levels happened in more extensive production systems; conversely, this result was not confirmed by Chobtang et al. [53] or by Morais et al. [54]. However, usually, the greater the productive level of the farm, or its productive efficiency, the lower the environmental footprint per kg of milk [55]. Moreover, Price and Bell [56] observed that the improvement of the genetic selection practices of dairy cows in Australia in the last 10 years led to a reduction in CO_2_eq emissions of about 1%, and Wattiaux et al. [57] explained that the factor most associated with farms’ emissions was the management practices. It can be argued that the emissions values obtained in the present study are in line with those reported in most studies considering farms with similar production efficiency, but irrespective of the breed reared. Different considerations could be drawn if the m^2^ of UAL was considered as the functional unit instead of milk. In fact, in comparison to the extensive mountain dairy farms considered by Salvador et al. [22], the present study showed a lower environmental footprint per kg of FPCM—1.43 vs. 1.46 kg CO_2_eq—but higher per m^2^ of UAL—1.64 vs. 0.69 kg CO_2_eq. Bava et al. [58] explained that the environmental footprint per ha can be positively related with the intensification of the productive process. The carbon footprint per kg of FPCM was 1.12 ± 0.085 and 1.23 ± 0.096 kg CO_2_eq when using a physical and economic allocation, respectively (Figure 2). These values were lower by 22% and 14% than that obtained without allocation. Kiefer et al. [21], adopting the physical allocation procedure, showed a reduction in the emissions allocated to milk by 19% in farms that mainly reared Holstein animals and 24% in farms that mainly reared animals of the dual-purpose Simmental strain. These data show how the method of physical allocation, which also considers the co-products to milk production, is particularly relevant in farms rearing dual-purpose breeds. The average on-farm contribution to GWP was 68.7% (data not shown) while the contributions of different emissions sources are shown in Table 2. Enteric emissions and manure storage together represented, on average, 56% of total GHG emissions. From this point of view, the most important gas is CH_4_; therefore, maximizing the synthesis efficiency of ruminal microbial fermentations can be considered a possible strategy to reduce the emission of gases that contribute to GWP. The other main contribution to GWP is represented by off-farm emissions related to the production of feed purchased by farms, constituting 20% of the total emissions.

As reported in Figure 2, considering AC without allocation and in the BASE scenario, the emission of SO_2_eq/kg FPCM was 18.99 ± 1.408 g. This value falls within the range of emissions calculated by Guerci et al. [51]—7.4–25.6 g SO_2_eq/kg ECM—and is very similar to the value obtained by Cederberg and Mattsson [59]—18.0 g SO_2_eq/kg ECM—in intensive dairy farms. The acidification footprint per m^2^ of UAL was 21.69 ± 1.764 g SO_2_eq (data not shown). However, even in this case, if we compare the results of the present study with those obtained by Salvador et al. [22], who considered dairy cows of dual-purpose breeds but in more extensive husbandry systems, the AC values are lower if referring to the kg of FPCM—19.0 vs. 27.2 g SO_2_eq—but higher, if referring to the m^2^ of SAU—21.7 vs. 12.4 g SO_2_eq. The acidification footprint per kg of FPCM was 14.84 ± 1.091 and 16.22 ± 1.246 g SO_2_eq using physical and economic allocation, respectively (Figure 2). These values were lower by 22% and 15% than that obtained without allocation. On average, 90% of the emissions related to acidification potential derive from on-farm activities (data not shown). It is clear that, for this category of emissions, the farm’s own activities have a crucial role. As reported in Table 2, 75% of the emissions are derived from the management of manure, which, together with crop production, represent about 90% of the total emissions. This percentage falls within the range found by Guerci et al. [51]—83–96%. These high values can be explained when considering that the main thing responsible for acidification in dairy livestock systems is the volatilization of ammonia that occurs during the storage, treatment and distribution of manure. In fact, according to the study by Cederberg and Mattsson [59], almost 90% of the acidification potential would be due to ammonia losses.

Considering EUP without allocation and in the BASE scenario, the emission of PO_4_^3−^eq/kg FPCM was 6.36 ± 0.486 g (Figure 2). Guerci [51] reported EUP values between 4.6 and 11.1 g PO_4_^3−^eq/kg FPCM. Bava et al. [58], considering dairy farms in the Po Valley with an average area of 41 ha and an average consistency of 90 cows, and Guerci et al. [60] obtained values higher than those of the present study—7.3 and 9.0 g PO_4_^3−^eq/kg FPCM, respectively. The eutrophication footprint per m^2^ of UAL was 7.10 ± 0.394 g PO_4_^3−^eq (data not shown). Guerci et al. [60] calculated that the incidence of on-farm emissions compared to the total was 74–78%, a value only slightly lower than the 82% obtained by the present study. It is interesting to note that, in the farms considered, the EUP is mainly due to manure management (45%) and to crop production (37%) (Table 2), and the gas mainly responsible is NH_3_, which develops during the manure management and nitrogen losses on land, in particular as N leaching. The incidence of emissions linked to the production of the feed purchased is 13%, which also can be considered relevant. Therefore, the improvement of the efficiency of the use of concentrates in feeding and the equilibrate use of organic and mineral fertilizers in soils can be considered valid strategies to reduce the environmental footprint, in terms of EUP, of the farms.

As previously reported, the environmental footprint of the farms was mainly due to CH_4_ and NH_3_ emissions and N leaching in the soil. These emissions were mainly assessed with a Tier 2 method, which leads to some uncertainty in the results, as it only includes country-specific emission factors. Indeed, to improve the reliability of the results, a Tier 3 method should be used; however, in the case of the present study, it was not possible because of the absence of emission factors disaggregated at a sub-national level. The development of appropriate field and laboratory methods could contribute to overcoming this problem, e.g., the use of rumen continuous fermenters [61] could allow one to obtain a reliable assessment of the rumen methane emissions of each single farm.

**Figure 3:**
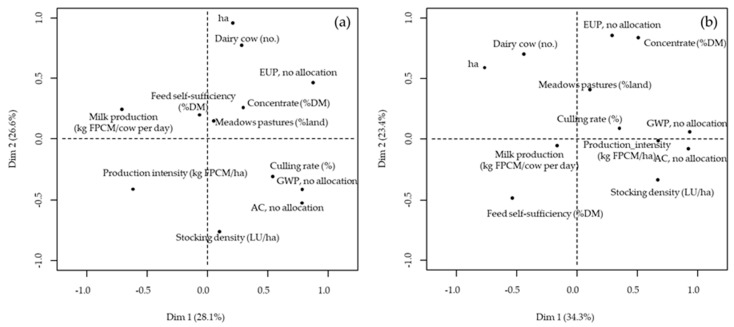
Principal component analysis of farm characteristics and Global Warming Potential (GWP), Acidification potential (AC) and Eutrophication potential (EUP) expressed as CO_2_eq per kg of Fat Protein Corrected Milk (FPCM) (**a**) or as CO_2_eq per m^2^ of Utilizable Agricultural Land (**b**). No allocation method was considered.

Figure 3a shows the PCA of farm characteristics and GWP expressed as CO_2_eq/kg FPCM calculated with the no allocation method. The first and the second principal component explained 28.1% and 26.6% of the total variance, respectively. All the impact categories considered—GWP, AC and EUP—were characterized by positive loadings on the first component; conversely, on the same component, milk production per cow and production intensity were characterized by negative loadings. This means that, as previously discussed, the environmental emissions of the farms could be reduced by increasing milk production per cow and production intensity. Interestingly, the emissions, especially in terms of GWP and AC, were closely associated with the culling rate, highlighting the importance of improving the efficiency, not only in milk production performances, but also in the reproductive performances of the animals, for reducing the environmental footprint of the farms. The results change using UAL instead of FPCM as the functional unit (Figure 3b), as also explained by Bava et al. [58]. Indeed, in this case, GWP and AC were positively associated with production intensity. Moreover, the first principal component, which explained 34.3% of the total variance, highlighted a negative relationship between the impact categories GWP and AC and the feed self-sufficiency of the farms, while the second principal component, which explained 23.4% of the total variance, highlighted a negative relationship between EUP and feed self-sufficiency and a highly positive relationship between EUP and the percentage of the concentrates in the diets of animals. 

### 3.2. Environmental Sustainability and Allocations in Alternative Scenario Analysis (BEEF and HEMP Scenario)

Considering the BEEF scenario without allocation, the GWP, AC and EUP were 1.58 ± 0.111 kg CO_2_eq/kg FPCM, 20.75 ± 1.475 g SO_2_eq/kg FPCM and 6.86 ± 0.513 g PO_4_^3−^eq/kg FPCM, respectively (Figure 2). These values dropped by approximatively 36% and 23% with physical and economic allocation in comparison with the no allocation method, respectively (Figure 2). These results are in close agreement with the findings of Flysjö et al. [62], who calculated a reduction in the emissions for milk of about 23–37% in a production system similar to that hypothesized in the present study. Conversely, Zehetmeier et al. [63] reported higher reduction values (46–77%), and these differences could be due to the different breeds reared, as also shown by Kiefer et al. [21]. As expected, with the no allocation method, the BEEF Scenario had higher emissions compared to the BASE Scenario, both in terms of GWP, AC and EUP (+8–10%) (Figure 2). However, considering physical allocation and, hence, also the co-product beef in the BEEF Scenario, GWP, AC and EUP were 1.01 ± 0.065 kg CO_2_eq/kg FPCM, 13.22 ± 0.737 g SO_2_eq/kg FPCM and 4.42 ± 0.338 g PO_4_^3−^eq/kg FPCM, respectively, and were reduced in respect to the BASE scenario by 10–11%. Also, other studies [21,22] observed that fattening the calves that exceed the culling rate can be a suitable tool for reducing the environmental footprint of farms. In particular, Salvador et al. [22] calculated a reduction in environmental footprint by 3–6% related to milk if the calves of dual-purpose breeds were fattened directly on the farm. However, with the economic allocation method, the reduction of emissions in the BEEF scenario in respect to the BASE scenario were more modest, reaching values of 0%, 0.6% and 2.0% for GWP, AC and EUP, respectively (Figure 2). Hence, these results indicate that increasing the multi-functionality of farms and the economic value of beef obtained in dairy farms can be favorable from the environmental footprint point of view.

Considering the HEMP scenario in comparison with the BASE Scenario, the GWP, AC and EUP dropped by approximatively 4.2–4.9%, 1.2–1.3% and 3.3–3.7%, respectively (Figure 2), depending on the allocation method adopted. These effects were mainly due to the reduction in the soybean or of the protein concentrates purchased (188 kg/LU on average per farm), and also by the reduction in other off-farms inputs, such as fertilizers and pesticides (85 kg/ha on average per farm ), as hemp is a low-input cultivation, which is explained by Amaducci et al. [45].

## 4. Conclusions

The environmental footprint of 1 kg of FPCM, based on the allocation method adopted, ranged from 1.1 to 1.4 kg CO_2_eq for GWP, from 14.8 to 19.0 g SO_2_eq for AC and from 5.0 to 6.4 g PO_4_^3−^eq for EUP. The emissions were positively related to culling rate and negatively related to milk production per cow and to production intensity. This means that improving the production and reproductive efficiency of animals can be relevant for reducing the environmental footprint of farms. Moreover, the fattening of calves on farms that already rear dual-purpose breeds and the cultivation of low-input crops, such as hemp, for using its by-products in animal feeding can be evaluated as successful strategies for reducing the environmental footprint of milk when a physical or economic allocation is considered.

## Figures and Tables

**Table 1:** Main characteristics of dairy farms (n = 11).

Item		Mean	SE
Land			
Farm land (ha)		59.7	8.63
Meadows and pasture (% land)		34.8	6.14
Maize and Sorghum land for silage (% land)		11.7	2.36
Herd			
LU total (no.)		124.9	14.18
Dairy cow (no.)		77.5	9.47
Milk yield (kg FPCM/cow per day)		23.5	1.57
Production intensity (kg FPCM/ha)		11,537	770.4
Culling rate (%)		29.6	1.21
Feed			
Feed efficiency (kg FPCM/kg DMI per cow)		1.08	0.07
Feed self-sufficiency (%)		76.0	5.43
Energy			
Diesel fuel use (kg/LU per year)		88.0	8.10
Electric energy use (kWh/LU per year)		202.4	47.61

LU = Livestock Units; FPCM = Fat and Protein Corrected Milk; DMI = Dry Matter Intake.

**Table 2:** Contribution (%) to global warming, acidification and eutrophication of different sources in BASE Scenario.

Source		Mean	SE
Global warming			
Manure and enteric emission		55.9	2.79
Crops		7.7	0.79
Feed purchased		20.0	2.83
Energy and fuel		5.9	0.60
Others		10.6	1.77
Acidification			
Manure and enteric emission		74.7	1.49
Crops		15.5	0.88
Feed purchased		5.4	0.68
Fertilizers		3.5	0.71
Energy and fuel		0.5	0.04
Others		0.4	0.07
Eutrophication			
Manure and enteric emission		45.2	3.28
Crops		36.7	3.34
Feed purchased		13.4	1.84
Fertilizers		4.0	0.83
Energy and fuel		0.2	0.03
Others		0.5	0.08
